# Machine learning in the loop for tuberculosis diagnosis support

**DOI:** 10.3389/fpubh.2022.876949

**Published:** 2022-07-26

**Authors:** Alvaro D. Orjuela-Cañón, Andrés L. Jutinico, Carlos Awad, Erika Vergara, Angélica Palencia

**Affiliations:** ^1^School of Medicine and Health Sciences, Universidad del Rosario, Bogotá, Colombia; ^2^Biomedical Engineering, Universidad Antonio Nariño, Bogotá, Colombia; ^3^Subred Integrada de Servicios de Salud Centro Oriente E.S.E, Bogotá, Colombia

**Keywords:** tuberculosis diagnosis, machine learning, relevance analysis, machine learning in the loop, diagnosis support systems

## Abstract

The use of machine learning (ML) for diagnosis support has advanced in the field of health. In the present paper, the results of studying ML techniques in a tuberculosis diagnosis loop in a scenario of limited resources are presented. Data are analyzed using a tuberculosis (TB) therapy program at a health institution in a main city of a developing country using five ML models. Logistic regression, classification trees, random forest, support vector machines, and artificial neural networks are trained under physician supervision following physicians' typical daily work. The models are trained on seven main variables collected when patients arrive at the facility. Additionally, the variables applied to train the models are analyzed, and the models' advantages and limitations are discussed in the context of the automated ML techniques. The results show that artificial neural networks obtain the best results in terms of accuracy, sensitivity, and area under the receiver operating curve. These results represent an improvement over smear microscopy, which is commonly used techniques to detect TB for special cases. Findings demonstrate that ML in the TB diagnosis loop can be reinforced with available data to serve as an alternative diagnosis tool based on data processing in places where the health infrastructure is limited.

## Introduction

Artificial intelligence (AI) is a set of bioinspired algorithms that are used to solve problems in different applications. Within this wide area, machine learning (ML) is a common subfield in which models learn from examples of data, taking advantage of the idea of adjusting parameters in classification or regression tasks (1). There are several different ML models according to the fundamental concepts for adapting the parameters, with diverse examples including naive Bayes, decision or classification trees, support vector machines (SVM), and artificial neural networks (ANNs), which emulate the behavior of the brain through connectionist models. Besides these and other ML models, new models are continuously being proposed (2).

Tuberculosis (TB) is a disease caused by the *Mycobacterium tuberculosis* bacillus, and the World Health Organization still considers it a global emergency because of its high estimate of more than 1.4 million fatalities in the last 3 years (3). In developing countries, TB incidence is as high as 282,000 new cases in recent years with a mortality rate of 2.4 per 100,000 populations. In one specific place, Colombia, the reported TB incidence was 33, the prevalence was 48, and the mortality was 1.6 per 100,000 populations. Given these numbers, any contribution to decreasing TB fatalities is welcomed. *M. tuberculosis* is slow-growing and replicates itself every 24 h, an important fact that determines subacute symptoms. Additionally, the main organ affected by TB is the lung, and because of this, the main signs of the disease are respiratory-related (3). Coughing and expectoration allow for assessing the probability of TB by studying sputum; however, because TB is an infectious disease, the accurate diagnosis is microbiological (4).

In the health area, AI has been applied to solve problems in public health, medical images analysis, and diagnosis support systems (5–8). For TB, different approaches have been proposed since 1999 with the work of El-Solh et al. (9), for whom medical images were the main source of information. Advances in this field have allowed for better detecting thoracic diseases including TB, pneumonia, asthma, and cancer (10, 11). Investigators have widely used specific ML models in health systems to contribute to improving TB diagnosis by taking advantage of available meaningful data (12, 13), such as data from clinical information (14–16), or molecular biology (17, 18).

ANNs have been particularly valuable in incorporating ML into TB diagnosis through different architectures such as multilayer perceptrons (MLP), self-organizing maps, and adaptive resonance theory (ART) joined to fuzzy models in the Fuzzy-ART approach to support detection and clustering in risk groups for pulmonary TB (19–21) and pleural TB (22–24). Researchers have used different data sources to support health professionals in daily tasks such as collecting breathing acoustic signals (25) and other clinical variables (20, 26).

Finally, TB researchers have used deep learning (DL) architecture using vast data sets to provide scenarios based on images (27–29). For instance, one important task was establishing the *ImageCLEF* data set, which allowed users to determine TB type and treatment resistance using coaxial tomography images (28, 30); researchers have also used images from radiography to support health professionals' decision making (31–33). Generally, DL has been widely applied in assisting with medical diagnosis, utilizing radiography images, and obtaining highlight results (34, 35). Additionally, one DL subfield, transfer learning, entails refining large pretrained models with new data, and several researchers have applied transfer learning to the same kinds of medical images (27, 36).

Nevertheless, despite its demonstrable benefits, ML's effectiveness can be limited by data availability constraints related to inadequate information technology infrastructure. Precarious health systems that cannot or do not collect radiographic information or conduct specialized testing significantly complicate the implementation of ML models. Researchers have analyzed these characteristics and proposed infrastructure for developing regions that can accommodate few variables and poor information systems have been treated for developing regions (19, 21).

The present work proposes ML techniques as a tool in the loop of TB diagnosis, where health professionals make decisions but with extra help based on limited available data. This scenario is studied for using ML in situations with limited infrastructure for application within the complete TB diagnosis protocol.

## Machine learning in the loop

The concept of the “algorithm-in-the-loop” is related to the use of ML models to support decision making and improve both human–computer interactions and human performance (37). Interaction between the model and users in a loop is not limited to simple representations of performance such as numbers but extends to a global idea that articulates ethics, policies, and standards (38). Including AI and ML stages in the clinical decision making support workflow can ultimately improve patient experiences and outcomes and optimize health system performance (8). Interactive ML is another term for when algorithms and humans work together to improve the results in terms of metrics, understandability, and outcomes (39).

For the case of TB, diagnosis was long based on respiratory symptoms followed by testing suspicious patients with a serial sputum smear; however, although this test is simple, it is necessary to consider some aspects in determining its usefulness. Smear microscopy is performed using sputum smear and staining that allows direct microscopic visualization of the bacillus. However, diagnostic sensitivity is low, around 60%, because a high number of microorganisms per cubic millimeter of a sample is required to obtain results (40). Indeed, a high percentage of people with the disease cannot be diagnosed using this method, and furthermore, detected bacillus could be a non-TB *mycobacterium*. A more sensitive assay is a culture in either solid or liquid medium, which needs at least 2 weeks to obtain results (41). Following more recent advances, molecular testing is now available: Polymerase chain reaction (PCR) identifies the TB bacillus with high sensitivity and in approximately 2 h (42). However, the infrastructure for this technology is limited in developing countries such as Colombia.

From the ML point of view, different applications have particular characteristics such as requiring biomedical data that have high uncertainty and incompleteness (43), and strategies beyond straightforward ML are sometimes demanded. For the present study, ML in the loop (MLL) is investigated; this strategy depends on how the ML tool will be used. Researchers have analyzed the necessary workflows to improve results (44), but in medicine, where health professionals play an indispensable role, other investigators have studied the doctor-in-the-loop in terms of system performance (45, 46). Today, how ML models perform is no longer the sole concern; models' generalizability and functionality during human interaction are also important. Assessing these broader aspects of performance allows for understanding important aspects of decision making and operation that must be considered in system designs (47).

**Figure 1:**
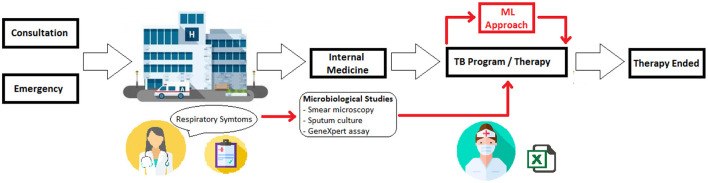
Schematic of using ML in TB diagnosis. During the TB diagnosis, ML tools are employed to support the decision about the antituberculosis therapy beginning.

Figure 1 depicts the MLL process for TB diagnosis support that was studied for the present work. First, a subject with respiratory disease symptoms arrives at the medical center for either a consultation or an emergency. There, a member of the medical staff examines the possible patient and then sends the patient to internal medicine for a more detailed examination. After this deeper analysis, if the patient's respiratory symptoms continue, medical staff request three main exams to detect pulmonary TB: sputum smear microscopy, sputum culture, and molecular assay (GenXpert®). If results from these three exams indicate infection, the patient begins antituberculosis therapy. Meanwhile the results are definitive, there is no positive diagnosis. However, the patient initialize the antituberculosis treatment. It is at this point where ML was applied to assist the medical staff members in diagnosis.

At the study facility, the health care workers are responsible for acquiring basic patient information equivalent to the medical records obtained in other stages. This information is input into a registry for the use of the institution's TB program; the protocol to detect TB can be time-consuming, and using ML with this registry could expedite diagnosis. This study proposed to apply MLL searches to support health care workers during the time the test results take. This allows staff to efficiently manage patient treatment according to the need for isolation, hospital capacity, and necessary medications.

## Materials and Methods

### Data set

Data were acquired through the TB program at *Hospital Santa Clara* (HSC) in Bogotá D.C., Colombia. The HSC is an important public institution associated with the *Subred Integrada de Servicios de Salud Centro Oriente* (SCO, Middle East Subnetwork of Health Services) that treats vulnerable populations with low socioeconomic status or high risk of sexually transmitted infections as well as persons who live in overcrowded conditions.

As explained earlier, the data were collected within the hospital's traditional TB diagnosis process. Information was considered from 233 clinical suspected pulmonary TB subjects whose data had been acquired in the period from January 2017 to December 2019. From this set, 184 subjects (79%) had TB confirmed and 36 subjects (15%) were determined to be disease-free based on smear microscopy, culture, and molecular examination following the national protocol to diagnose TB (48). Thirteen subjects were not considered because they had no available information on their TB status. The Ethics and Research Committee of the SCO approved this study on the basis of the use of anonymous data with only population-related variables that posed no risks to subjects. Informed consent was not required because all data were retrospective and anonymous.

At the HSC, electronic health records are used, but they are not standardized across the country; records can include diagnoses and symptoms of medical conditions such as diabetes, chronic kidney disease, and immunosuppression such as by the human immunodeficiency virus (HIV). Sociodemographic variables are also important for TB diagnosis (49), and the SCO commonly treats vulnerable populations such as persons who are indigenous, homeless, migrants, or refugees for TB. Although some of the data are available, the different information systems do not always communicate with each other. For this reason, only the variables that were available at the beginning of the TB program were applied for this study, as specified above. Using only these data allowed for simulating a scenario with limited information.

Health care workers at this point of TB diagnosis collect only seven variables, which were the ones considered in the present work: sex, age, type of population, city location, HIV/AIDS (acquired immunodeficiency syndrome) status, antiretroviral treatment status, and the number of days since treatment onset (see Table 1). Age and number of days were discrete numeric variables that were normalized by maximum of 100 and 15, respectively. Sex was a binary variable where a patient was either male or female, and this variable was set at 00 when no data were available. HIV and antiretroviral treatment status could take either of three possible values: positive, negative, or unknown. Finally, the type of population and city location were, respectively, coded with zeros and ones to reflect if a clinic visitor was a member of a specific vulnerable group and where in Bogotá City the client resided based on established geographic divisions.

**Table 1:** Variables collected.

**Variable**	**Values**
Sex	Male
	Female
Age	Numeric: 0–100
Type of population	Homeless
	Native
	Exile
	Immigrant
	Prison
	Violence Victim
	Other
City location	Antonio Nariño
	Barrios Unidos
	Bosa
	Chapinero
	Ciudad Bolívar
	Engativá
	Fontibón
	Kennedy
	La Candelaria
	Los Mártires
	Puente Aranda
	Rafael Uribe Uribe
	San Cristóbal
	Santa Fe
	Suba
	Teusaquillo
	Tunjuelito
	Usaquén
	Usme
	Out of Bogotá City
	Unknown
HIV/AIDS status	Yes
	No
	Unknown
Antiretroviral treatment status	Yes
	No
	Unknown

### Machine learning models

ML models are a set of algorithms that learn from data (50). For the present study, four MLL models were compared for their usefulness to health professionals and for the interactions between available features in the TB decision making process. In health sciences, logistic regression (LR) algorithms are widely applied to associate predictors or input variables to an output that represents a detection or estimation of the illness (41, 51). To evaluate the present scenario, LR was the fifth model considered to determine the possible contribution of traditional tools. The optimization algorithm was based on a quasi-Newton method, the Broden–Fletcher–Goldfarb–Shanno (*lbfgs*) approximation; additionally, penalization was used with a maximum of 100 iterations.

Classification or decision tree (DT) algorithms are trained through supervised learning and are considered a non-parametric method for classification or regression (52). DT structure is based on nodes and leaves, where each node is represented by a function that divides the information flow into two or more classes according to the function's output. For the present case, this function was based on the Gini coefficient. A notable advantage of this ML model is that it allows for visually determining the conditions for the input variables and the leaves. Random forest (RF) is a special DT model, in which more tree structures are analyzed and tested (53, 54). Then, the best configuration of trees is selected for the classification or regression, according to a sample from the data set and avoiding model overfitting.

SVMs deal with the boundary between hyperplanes that divides the data classes from input variables represented in a features space (55, 56). The hyperplanes are built from support vectors obtained from the training data and optimized according to the support vectors with the best performance. This model is widely applied with kernelling, modifying the initial non-linear separable space into a linear separation through a non-linear kernel that for the present case was Gaussian.

Finally, an MLP was applied as a model to detect the TB cases because the results were known in this specific problem (57). For this case, an architecture with one hidden layer was trained to detect TB. The number of input nodes was equal to the number of variables, and there was one output node. Resilient backpropagation was applied for training and stop criteria with a maximum of 500 epochs, zero gradients, and early stopping, the first time early stopping was considered.

Cross-validation was conducted to assess the performance and generalization of the models (58). Based on the special scenario under study, the mode of data acquisition, and the possibility of a system application in the future, the data were divided into three sets. This allowed for establishing the models based on 2 years of data that were validated and tested for generalizability in the third year. Through this process, the tool can be used using previous information with similar properties. Table 2 shows these sets, the year of acquisition, and the number of instances per set.

**Table 2:** Sets used for cross-validation.

**Set**	**Year**	**TB positive**	**TB negative**	**Total**
1	2017	34	9	43
2	2018	52	22	74
3	2019	55	10	65
Total		141	41	182

A process to balance the classes was implemented, searching to adjust the inequality between positive and negative TB for the classes. In this case, a weighted training process of internal parameters for each model was regulated according to the frequency of the instances by class (59).

### Variable analysis

Study variables were analyzed through the performance computation for each ML model under study. The variables in Table 1 were converted to zero and then applied to the best trained of the DT, LR, RF, SVM, and MLP models. Subsequently, model performance metrics such as accuracy, sensitivity, and specificity were compared.

### Automated machine learning

Automated ML (aML) was also tested to find the best models (60), and the Tree-based Pipeline Optimization Tool (TPOT) was applied to obtain the best detectors (61). This was carried out because of differences in the ML models' performance. Here aML and TPOT were used to compare the individual models' performance and to determine the influences of the ML model parameters in the search results.

## Results

Table 3 shows the findings for the training process and the test scores with data from the year left out in the cross-validation described before; accuracy (ACC), sensitivity (SE), and specificity (SP) were collected to determine the differences due to the balance between positive and negative TB for each year (see Table 2). Additionally, the area under the receiver operating curve (AUC) allowed for considering SE and SP simultaneously.

**Table 3:** Results for the ML models.

**Model**	**Validation year**	**Training**				**Test**			
		**Accuracy**	**Sensitivity**	**Specificity**	**AUC** ^*^	**Accuracy**	**Sensitivity**	**Specificity**	**AUC** ^*^
DT	2017	0.75	0.82	0.50	0.65	0.70	0.82	0.22	0.53
	2018	0.94	1.00	0.73	0.86	0.68	0.81	0.36	0.59
	2019	0.97	1.00	0.91	0.96	0.72	0.75	0.60	0.68
RF	2017	0.81	0.83	0.72	0.73	0.70	0.79	0.33	0.60
	2018	0.94	0.94	0.89	0.87	0.70	0.87	0.32	0.63
	2019	0.89	0.90	0.87	0.85	0.82	0.85	0.60	0.77
LR	2017	0.63	0.59	0.78	0.63	0.63	0.59	0.78	0.61
	2018	0.71	0.71	0.68	0.63	0.65	0.73	0.45	0.62
	2019	0.62	0.58	0.74	0.63	0.65	0.60	0.90	0.84
SVM	2017	0.99	0.98	1.00	0.97	0.65	0.74	0.33	0.45
	2018	0.94	0.92	1.00	0.86	0.61	0.75	0.27	0.56
	2019	0.89	0.86	0.97	0.85	0.68	0.69	0.60	0.68
MLP	2017	0.82	0.95	0.38	0.77	0.74	0.88	0.22	0.65
	2018	0.87	1.00	0.26	0.93	0.74	1.00	0.14	0.65
	2019	0.79	0.99	0.23	0.83	0.85	0.93	0.40	0.82

* *AUC, Area Under Receiver Operative Curve*.

The LR, RF, and MLP models achieved the best results, obtaining the highest AUC, 0.84, in the test set (see Table 3). This value can be compared with the maximum AUC of 0.96 in the DT model for the training set, demonstrating that it was difficult to generalize the findings from the present application.

Table 4 presents the ACC, SE, SP, and AUC means and standard deviations for the three test data subsets. The table shows that MLP obtained the best results for ACC, SE, and AUC and that SP was the best with the LR model. These findings suggest that combining models might give better results for these metrics. Nevertheless, although SP was the best with the LR, that model had the worst results for ACC and SE, which suggests this model's suitability for the objective task of finding negative TB cases. Finally, the SVM model gave the worst results for most metrics.

**Table 4:** ML model results for the three test subsets.

**Model**	**Accuracy**	**Sensitivity**	**Specificity**	**AUC** ^*^
DT	0.70 ± 0.040	0.79 ± 0.001	0.39 ± 0.037	0.60 ± 0.005
RF	0.74 ± 0.069	0.83 ± 0.001	0.42 ± 0.025	0.67 ± 0.008
LR	0.64 ± 0.011	0.64 ± 0.006	0.71 ±0.054	0.69 ± 0.017
SVM	0.64 ± 0.001	0.72 ± 0.001	0.40 ± 0.030	0.56 ± 0.013
MLP	0.77 ±0.004	0.93 ±0.003	0.25 ± 0.017	0.71 ±0.009

* *AUC, Area Under Receiver Operative Curve. The bold values are the highest values for each column*.

**Figure 2:**
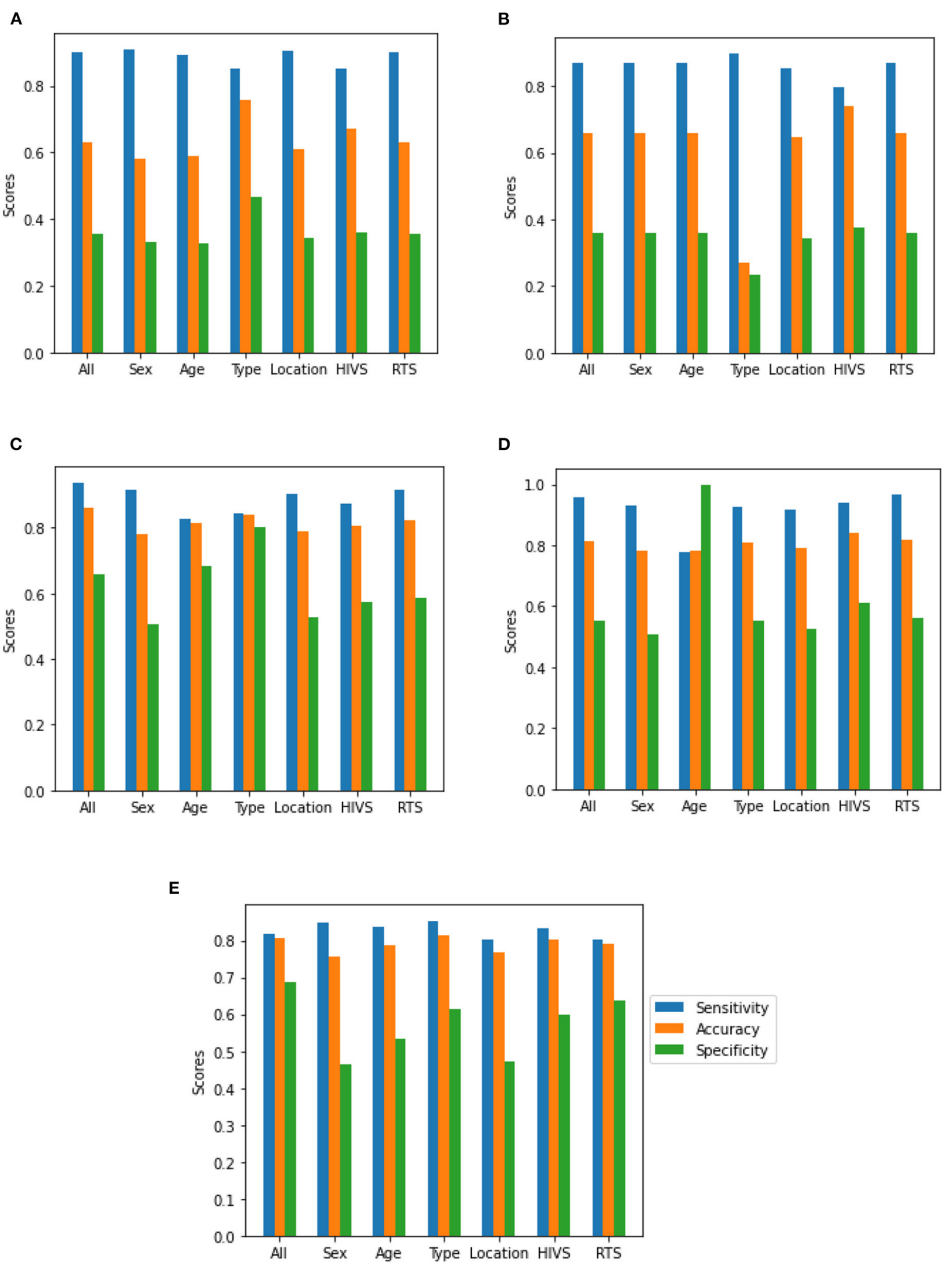
Sensitivity, accuracy, and specificity for all five ML models: **(A)** Logistic regression; **(B)** Classification tree; **(C)** Random forest; **(D)** Support vector machine; **(E)** Multilayer perceptron neural network. For all ML models is visualized the effect of using or not each one of the considered variables in terms of sensitivity (blue), specificity (green) and accuracy (orange). There it is possible to see how the metrics change, according to the inclusion or exclusion of the seven variables.

Table 5 presents the best results for each metric for all the studied models and the full data set, showing that the LR model had the best accuracy, SVM had the best sensitivity, and MLP had the best specificity. Additionally, following subsection 3.3, all models were checked for relevance. Specifically, for each model, the input variables (see Table 1) were set at 0, and then, ACC, SE, and SP were computed. Figure 2 shows the effect of this processing, notably that type of population was not important in the LR, RF, and MLP models; when the zero values were eliminated, the models' performance improved. Figure 2D shows that age caused significant differences in the SVM model. Finally, all variables were relevant in the MLP model.

**Table 5:** Best ML model results for the applied metrics and the full data set.

**Model**	**DT**	**RF**	**LR**	**SVM**	**MLP**
Accuracy	0.63	0.66	0.86	0.81	0.80
Sensitivity	0.90	0.87	0.94	0.95	0.82
Specificity	0.35	0.36	0.66	0.55	0.68

*The bold values are the highest values for each column*.

Table 6 presents the findings from testing aML and TPOT, which require less intensive user exploration of the hyperparameters. The table shows that the automated ML was more successful than manual exploration (see Table 3), although the results were similar. The first model, for the year 2019, applied six ML models: two passive-aggressive, two MLPs, one extra tree, and one gradient boosting. The second model, for 2018, had 28 models that included a number of the different strategies presented here (e.g., MLP, RF, and logistic regressors). For the 2017 case, aML produced a combination of five models (two random forests, one mlp, one passive-aggressive, and one stochastic gradient descent). Table 7 presents the aML and TPOT results for all 3 years. Specificity is considerably affected in this automatic generation of models, which is ineffective and not appropriate in the context of diagnosis support.

**Table 6:** Results for the auto ML models by year.

**Model**	**Validation year**	**Training**				**Test**			
		**Accuracy**	**Sensitivity**	**Specificity**	**AUC** ^*^	**Accuracy**	**Sensitivity**	**Specificity**	**AUC** ^*^
AutoML	2017	0.86	0.85	1.00	0.92	0.79	1.00	0.00	0.50
	2018	0.92	0.90	1.00	0.95	0.70	0.70	0.50	0.60
	2019	0.91	0.92	0.88	0.90	0.83	0.94	0.46	0.70
TPOT	2017	0.77	1.00	0	0.50	0.79	1.00	0	0.50
	2018	0.85	0.84	1.00	0.92	0.73	0.72	1.00	0.86
	2019	0.74	0.74	1.00	0.87	0.84	1.00	0.00	0.50

* *AUC, Area Under Receiver Operative Curve*.

**Table 7:** Results for the auto ML models for 3 years.

**Model**	**Accuracy**	**Sensitivity**	**Specificity**	**AUC** ^*^
AutoML	0.77 ± 0.004	0.88 ± 0.025	0.32 ± 0.077	0.60 ± 0.010
TPOT	0.78 ± 0.003	0.90 ± 0.026	0.33 ± 0.333	0.62 ± 0.043

* *AUC, Area Under Receiver Operative Curve*.

## Discussion

TB detection in earlier stages is important to prevent transmission of the disease. However, irrespective of when a patient is diagnosed, patients in the populations studied in this work must be kept in isolation because these patients tend not to maintain safe distances as they are being treated.

Because of the lack of specific clinical symptoms, it is difficult for physicians to diagnose tuberculosis, but meanwhile, patients require rapid isolation to prevent spreading the disease to others. Presumptive TB cases require further analysis, and tools for completing specific tasks could reduce the workloads of health professionals. ML and AI could be effective in this context while keeping decisions under the purview of the medical staff. Furthermore, in developing or low-income countries such as Colombia, ML and AI can extend the availability of health care to remote regions with limited infrastructure and few if any health care personnel.

There remain many challenges to applying ML and AI in the health informatics field, but doing so can contribute to easing burdens for clinical personnel; further testing of these applications in real-world settings will be highly beneficial. Furthermore, the coworking between health professionals and health care AI is a challenge. The American Medical Association calls for considering AI an augmentation to human intelligence rather than a replacement (62). Recent authors have reported on developing this kind of articulation with health professionals as the center of the entire strategy (12).

In this study, the high incidence rate in the analyzed data set was related to the stage of the diagnosis process, although despite this, it is possible to see that not all presumptive TB cases were ultimately diagnosed as positive TB. This indicates that the ML tool identified variables that were imperceptible to humans, which could help improve therapy management as well as increase the efficient allocation of clinical resources (time, professional staff, medicaments, space, etc.). However, it was determined in this study that the unbalance between positive and negative TB cases could be offer a difficulty of the ML models training (59). However, the RF, LR, and MLP models achieved similar results for SE and SP, consistent with earlier findings for MLP models (19, 21, 33, 55); these findings support RF, LR, and MLP as appropriate models for diagnosis support. In the present study, MLP had the best AUC metric, which exhibits best balance between SE and SP. Additionally, the proposed models can decrease the number of cases for which treatment begins without a confirmed diagnosis, which should decrease health system costs in time and other resources. Regarding aML and TPOT, finding the hyperparameters was not a dilemma, but the SP results were not as good as they were with other models. Furthermore, it is common for health informatics applications to have access to only small data sets or represent only rare events, and these conditions significantly reduce the accuracy of the results from aML approaches (60, 61).

Diagnostic algorithms have been incorporated into several national and international recommendations and guidelines for optimizing patient approaches. In the case of Colombia, health entities must notify the alert surveillance system of public health diseases, to epidemiologically monitor and clinically control TB to verify the success of the treatment. National TB registries allow for acquiring adequate global information on all the current clinical and sociodemographic aspects of TB as well as the success of the treatment strategies used.

In terms of limitations of the present study, there was a high incidence of TB in the data set, which could have induced bias in the analyzed data; addressing this will require more specific scenarios that involve clinical observation. Additionally, TB culture is considered the gold standard for diagnosis in some cases, especially when the infrastructure of GenExpert is not available. In this study, although the hospital database can only hold a limited number of patients, the HSC is an important center for TB treatment in Bogotá City; future researchers could incorporate data from more institutions that treat TB. Finally, researchers could incorporate more technical aspects such as including ensemble methods, combining different ML models, and considering more sophisticated models as the next steps.

## Conclusions

The findings of this study make it possible to conclude that sensitive ML algorithms can support TB diagnosis by considering the clinical features of the cases as well as medical and sociodemographic risk factors of the patients. TB continues to be a global leading cause of death, and challenges remain in identifying, treating, and containing the disease in several communities. The mycobacteria–host relationship can delay diagnosis for a host of reasons, as can limited clinical resources for diagnosis. Computational tools such as those studied here can support timely TB diagnosis and treatment.

## Data availability statement

The raw data supporting the conclusions of this article will be made available by the authors, without undue reservation.

## Author contributions

Conceptualization: AO-C and CA. Methodology, supervision, and resources: AO-C and AJ. Software, writing—original draft preparation, funding acquisition, and visualization: AO-C. Validation: AO-C, CA, EV, and AP. Formal analysis, investigation, and writing—review and editing: AO-C, AJ, CA, EV, and AP. Data curation: AO-C, CA, and AJ. Project administration: AJ. All authors have read and agreed to the published version of the manuscript.

## Funding

This research was funded by *Ministerio de Ciencia, Tecnologia e Innovación of Colombia*—Minciencias, grant number 123380762899.

## Conflict of interest

The authors declare that the research was conducted in the absence of any commercial or financial relationships that could be construed as a potential conflict of interest.

## Publisher's note

All claims expressed in this article are solely those of the authors and do not necessarily represent those of their affiliated organizations, or those of the publisher, the editors and the reviewers. Any product that may be evaluated in this article, or claim that may be made by its manufacturer, is not guaranteed or endorsed by the publisher.
